# The utility of mass spectrometry-based proteomic data for validation of novel alternative splice forms reconstructed from RNA-Seq data: a preliminary assessment

**DOI:** 10.1186/1471-2105-11-S11-S14

**Published:** 2010-12-14

**Authors:** Kang Ning, Alexey I Nesvizhskii

**Affiliations:** 1 Department of Pathology, University of Michigan, Ann Arbor, MI 48109, USA; 2 Center for Computational Biology and Medicine, University of Michigan, Ann Arbor, MI 48109, USA; 3 BioEnergy Genome Center, Qingdao Institute of BioEnergy and Bioprocess Technology, Chinese Academy of Sciences, China

## Abstract

**Background:**

Most mass spectrometry (MS) based proteomic studies depend on searching acquired tandem mass (MS/MS) spectra against databases of known protein sequences. In these experiments, however, a large number of high quality spectra remain unassigned. These spectra may correspond to novel peptides not present in the database, especially those corresponding to novel alternative splice (AS) forms. Recently, fast and comprehensive profiling of mammalian genomes using deep sequencing (i.e. RNA-Seq) has become possible. MS-based proteomics can potentially be used as an aid for protein-level validation of novel AS events observed in RNA-Seq data.

**Results:**

In this work, we have used publicly available mouse tissue proteomic and RNA-Seq datasets and have examined the feasibility of using MS data for the identification of novel AS forms by searching MS/MS spectra against translated mRNA sequences derived from RNA-Seq data. A significant correlation between the likelihood of identifying a peptide from MS/MS data and the number of reads in RNA-Seq data for the same gene was observed. Based on *in silico* experiments, it was also observed that only a fraction of novel AS forms identified from RNA-Seq had the corresponding junction peptide compatible with MS/MS sequencing. The number of novel peptides that were actually identified from MS/MS spectra was substantially lower than the number expected based on *in silico* analysis.

**Conclusions:**

The ability to confirm novel AS forms from MS/MS data in the dataset analyzed was found to be quite limited. This can be explained in part by low abundance of many novel transcripts, with the abundance of their corresponding protein products falling below the limit of detection by MS.

## Background

Mass spectrometry-based shotgun proteomics has become the method of choice for the identification and quantification of proteins from complex biological samples such as cell lines and tissues [1,2]. In a typical proteomics experiment, proteins of interest are digested into peptides using a proteolytic enzyme such as trypsin. Resulting peptide mixtures are separated by single or multi-dimensional chromatography coupled online to a tandem mass spectrometer used to sequence the peptides. The acquired MS/MS spectra are then searched against protein sequence databases such as RefSeq or International Protein Index (IPI) database using tools such as SEQUEST or X! Tandem to identify peptide sequences (reviewed in [2]). The list of identified peptides is then used to infer the identities of the protein present in the sample [3].

Recent developments in MS, peptide and protein separation chemistry, and computational methods for MS/MS data analysis have made high throughput proteomic characterization of complex biological samples feasible [4-6]. However, it has been observed that a significant number of high quality spectra in a typical dataset remain unassigned when searched against existing protein sequence databases [7,8]. Possible reasons for this include post-translational modification, either biological or chemical, as well as the presence of novel peptides corresponding to protein isoforms not included in the searched protein database [7-9]. At present, major protein databases typically used for MS analysis are incomplete with respect to AS variants predicted from genomic data in order to keep minimum redundancy [10]. Moreover, many of these protein isoforms are still not well annotated [9,11,12]. Translated EST (Expressed Sequence Tag) databases has been used for MS/MS search [9,13,14] to identify peptides supporting novel AS forms, or for verification of peptides identified by the 6-frame search that could not be aligned perfectly to known coding regions [15]. However, EST sequences are redundant [16] and contain many errors originated from cDNA clones [9,17,18]. Construction of the translated mRNA sequences based on all hypothetical AS forms in the genome [19,20] has also been attempted with some success previously.

Recently, next generation sequencing technique based on high throughput deep sequencing of complementary DNAs (RNA-Seq) has emerged as a powerful method for fast and comprehensive profiling of mammalian transcriptomes [18,21,22]. Studies using RNA-Seq have found evidence for many novel exons, and it was also shown that there were more AS forms than previously expected [12]. By creating translated mRNA sequences from RNA-Seq data, MS-based proteomic data can be used to identify the protein products of these novel exons and AS forms [23], thus providing protein-level validation for RNA-Seq derived gene models. However, it remains unclear how many of these novel AS forms could be identified from MS/MS spectra in practice. A far more fundamental question is how many of these novel splice forms are actually translated into functional protein products [24,25]. In this study, we perform a preliminary analysis using publicly available mouse tissue RNA-Seq data and MS/MS data generated on mouse mitochondrial proteome in same tissues, with a focus on the MS-based validation of novel AS forms.

## Methods

### Construction of translated mRNA sequence database

The first step for novel peptide identification is the generation of the protein sequence database from mRNA transcripts predicted by RNA-Seq. Novel mRNA sequences for novel AS forms are extracted by RNA-Seq followed by alignment of short reads with known gene models. Specifically, based on splice reads (junction reads) from RNA-Seq, novel mRNA sequences corresponding to AS are generated by connecting exons for which splicing events between them are identified. The quality of mRNA sequences is then analyzed by statistical analysis [12], and only high quality mRNA sequences are used. Translated mRNA sequence database is generated by translation of all open reading frames (ORFs) for these novel transcripts by 6-frame translation. To make translated mRNA sequences suitable for reliable MS/MS spectral searching, translated ORFs are required to be at least 30 amino acids long.

### Peptide identification via combined database search

**Figure 1:**
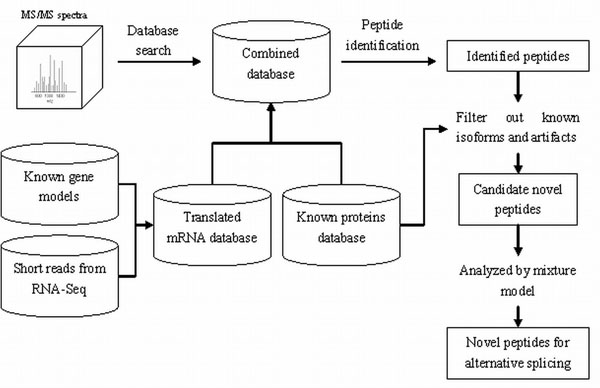
Overview of the computational strategy.

For novel peptide identification from translated mRNA sequences, we have adopted the combined database search approach: translated mRNA sequences are first appended to a commonly used protein database (mouse IPI database in this work). An equal size of reverse sequences were then generated and appended to the database as decoys for false discovery rate (FDR) analysis. All MS/MS spectra were searched against this combined database using X!Tandem [26] with k-score [27] scoring function, and using the following parameters: parent ion mass tolerance of ±100 ppm, monoisotopic mass, allowing tryptic peptides only, not more than one missed trypsin cleavage, and allowing two variable modifications: oxidation and N-terminal acetylation. The search results were then analyzed by PeptideProphet [3]. Spectra assigned to peptides with probability of 0.9 or greater (corresponding to an estimated FDR of less than 0.02) were considered assigned. Peptide identifications were additionally filtered to keep only those based on high quality MS/MS spectra (quality score above 1 according to QualScore [15]). Among these peptides, those that were present in the translated mRNA sequence database only (and not in the mouse IPI or mouse subset of NCBI nr database) were considered as candidate novel peptides. The overall search scheme is illustrated in Figure 1

## Results

### Experimental datasets

MS/MS data were taken from a recently published study on mouse mitochondrial proteome (MitoCarta dataset) [28], in which in total 1098 mitochondrial genes/proteins (NCBI Entrez genes [29]) were identified from multiple tissues. Mass spectra were generated using an LTQ-Orbitrap mass spectrometer. MS/MS data from three tissues, brainstem (282,360 spectra), liver (256,355 spectra) and skeletal muscle (215,668 spectra) were used in this study. Gene expression values were directly downloaded from http://woldlab.caltech.edu/rnaseq/. Essentially, these values were estimated from RNA-Seq data by RPKM (Reads Per Kilobase of exon per Million mapped reads). for these three tissues were obtained from Mortazavi *et al*. [18], which provided comprehensive information about novel exons and AS forms in the mouse genome. The mouse genome data mm9 is based on UCSC genome browser [30], obtained from the Build 37 assembly by NCBI and the Mouse Genome Sequencing Consortium, plus gene models annotated by UCSC and collaborators worldwide. To generate the translated mRNA sequence database, genome sequences that were identified by short reads as splice junctions (covered by at least one unique short reads) and were not in known gene models in mm9 were translated into protein sequences by 6 open reading frame translation. In doing so, 25 amino acids were taken from the two compatible exons to create junction amino acid sequences, and shorter junctions were filtered out. There were in total 573,053, 346,895 and 344,537 junctions for AS forms in the translated mRNA sequence database for brainstem, liver and skeletal muscle tissues, respectively. The combined database was created by appending the translated mRNA sequences to the IPI mouse protein sequences database v3.50 [10] (110,618 entries). Translated EST sequence database [9] (http://edwardslab.bmcb.georgetown.edu/, version 1.4, accessed 05/15/2009) was also used to search for novel peptides to compare with the result of searching translated mRNA sequences.

### In silico analysis of the prevalence of AS events that could be identified by tryptic peptides

Before searching MS/MS spectra against translated mRNA sequences, we have analyzed the prevalence of AS from mRNA sequences that could be identified by tryptic peptides. This analysis was performed *in silico* in which the number of tryptic peptides that could straddle the two exons was computed. This *in silico* analysis independent of actual short reads and MS/MS spectra used, and it is also independent of how translated mRNA sequences were created.

It was discovered that by mapping junction reads from RNA-Seq to gene sequences in mm9, most of known genes contained more than one AS junction (Table 1). This was true for all mouse genes, but also for the specific subset of mitochondrial genes that was the subject of the MS based analysis that produced the dataset of MS/MS spectra used in this work. However, it was also observed that less than 6% of all known AS junctions in mm9 could be identified by tryptic peptides straddling the intron-exon boundary and having length between 6 and 30 amino acids long - the range most compatible with MS/MS sequencing (numbers were similar for all tissues) (Table 1). Allowing one missed trypsin cleavage in peptides (identification of such peptides is less likely) would less than double this proportion. To further investigate this, the AS junctions were categorized into known (translated sequences corresponding to the junction regions were present in the IPI [10] or NCBI nr protein sequence database), and potentially novel events. It was observed that out of all AS predicted, close to 80% were known AS. The proportion of identifiable junctions was similar among known and novel AS forms.

**Table 1:** Average number of distinct junctions (AS) per gene for three different tissues in mouse, and average number of distinct junctions per gene in three different tissues that could be identified by tryptic peptides.

Tissue	Ave. unique junctions per gene (All)	Ave. unique junctions identifiable by tryptic peptides per gene (All)	Ave. unique junctions per gene (Mitochondrial)	Ave. unique junctions identifiable by tryptic peptides per gene (Mitochondrial)
Brainstem	4.00	0.27	5.30	0.29 (0.45)
Liver	2.44	0.16	4.91	0.27 (0.42)
Skeletal Muscle	2.42	0.16	4.57	0.24 (0.39)

Column 2 and column 3 were based on all junction reads identified by RNA-Seq for all NCBI Entrez genes. Column 4 and column 5 were based on junction reads by RNA-Seq for 1098 mitochondrial genes. Numbers in brackets were based on tryptic peptides allowing one missed cleavage.

We also determined the number of known splice junctions specifically for all 1098 mitochondrial genes annotated in MitoCarta (table 1). On average, there were ~ 5 junctions per gene. Combined with the estimate of the number of novel junctions given above (~20%), and the expected proportion that can be identified by tryptic peptides (~5%), one obtains an estimate of approximately 50 novel peptides from mitochondrial proteins that one may expect to identify from translated mRNA sequences based on RNA-Seq data. Since the number of mitochondrial proteins identified in each individual tissue (brainstem, liver or skeletal muscle tissues used in this work) was actually less than the MitoCarta compendium (700 on average for each tissue), the expected number is reduced to ~ 35 peptides. Counting co-purifying non-mitochondrial proteins also identified in this dataset brings the expected number of novel peptides back to ~ 50 (~80 allowing one missed cleavage). For known AS forms, the same estimation procedure predicts ~200 peptides (~300 allowing one missed cleavage).

### Results of searching MS/MS spectra against the combined sequence database

For each of the three tissues, the combined database was created by appending the unique translated mRNA sequences to the IPI mouse protein sequences database v3.50 [10] (see Methods). All MS/MS spectra were searched against this combined database by X!Tandem [26] followed by PeptideProphet analysis [23]. Peptides identifications with PeptideProphet probability equal or greater than 0.9 (corresponding to an estimated FDR = 0.02) were considered identified. This led to 54,906, 59,988 and 46,077 spectra to peptide assignments for brainstem, liver and skeletal muscle tissues, respectively. Counting peptide sequences supporting known AS junctions only, there were 494, 301, and 282 identifications for brainstem, liver, and skeletal muscle tissues, respectively. Note that these numbers are close to the expected numbers for known AS forms obtained using *in silico* estimates described above.

Because the rate of false positives among the candidate novel peptides is significantly higher (0.38) than that among known peptides, we applied a more stringent filtering threshold to achieve an FDR of 0.02 specifically for the subset of novel peptides. In addition, to further eliminate false positive identifications due to sequence homology [15], candidate novel peptides were searched against mouse subset of NCBI nr protein database by BLAST [31], and all peptides with less than 3 mismatches compared to known sequences were removed from subsequent analysis. Applying these stringent filtering steps, which we believe were warranted, resulted in the identification of only 2~3 peptide per tissue. These numbers of identified peptides supporting novel AS junctions is clearly smaller than what is expected based on the *in silico* analysis (50-80 peptides). Furthermore, most of the novel peptides that were identified from RNA-Seq data could also be identified by searching against existing translated EST sequence databases.

### Effect of mRNA abundance and the likelihood of MS-based peptide identification

**Figure 2:**
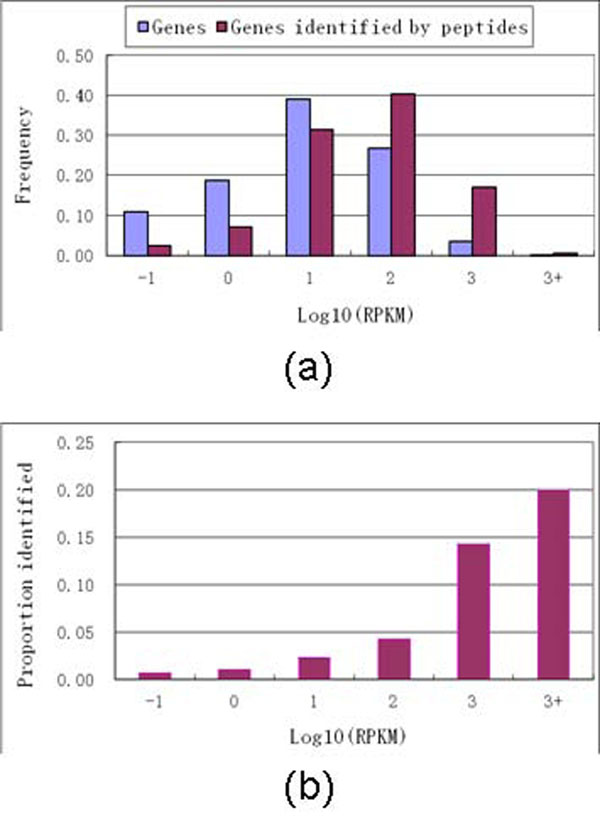
**Concordance between mRNA abundance and the proportion of genes that could be identified by peptides from MS/MS spectra.** (a) Histogram showing the normalized number (frequency) of all genes, and genes that are identified by peptides from MS/MS spectra, across the wide range of mRNA abundances (RPKM values, log scale). (b) Proportion of genes identified by peptides from MS/MS data. Data for brainstem tissue.

To gain a better understanding of why such a small number of novel peptides were identified from MS/MS spectra, we investigated the effect of mRNA abundance on the likelihood of protein identification. Based on all genes in the brainstem tissue dataset for which comparison could be made, we have used RNA-Seq based method (RPKM) [18] to measure gene expression levels. We then calculated what fraction of genes in a particular range of RPKM values that could be identified from MS/MS data. As shown in Figure 2, the higher was the level of gene expression (RPKM value), the more likely the corresponding proteins were identified from MS/MS data. The proportion of identified proteins dropped quickly with decreasing RPKM values.

A correlative analysis of RPKM values and protein abundances extracted from MS data using label-free quantification based on spectral counts [32,33] indicated a certain degree of correlation between mRNA and protein abundance levels (r=0.53) for mitochondrial genes/proteins in this dataset (N., Kang et al., in preparation). Thus, the trend shown in Figure 2 (b) reflects, indirectly via correlation between gene and protein abundances, the dynamic range limitation of MS-based proteomics. In other words, proteins present in the cell at lower abundance are being increasingly underrepresented in the lists of identified proteins. This suggests that the identification of peptides for novel exons or AS forms requires that the corresponding genes have a relatively high gene expression levels. The average lower abundance (short read count) of novel AS forms identified in RNA-Seq, as compared to that for known AS forms, may be one factor contribution to lower than expected rate of identification of novel junction peptides from MS/MS data.

### Efficiency of novel peptide identification process

All of the experiments were performed on a Linux server with 2.2 GHz CPU and 4.0GB RAM. The novel peptide identification process based on combined database was very efficient: the overall process of novel peptide identification from mass spectra for mitochondrial genes in brain tissue was within 3 hours, and most of the time was consumed by combined database search. Candidate novel peptide filtration based on searching known databases was fast due to the small number of candidate novel peptides.

## Conclusions

Deep sequencing by RNA-Seq allows comprehensive analysis of alternative splicing and identifies a large number of novel alternative splice isoforms. In this study, we have tried to use MS-based proteomic data for protein-level validation of these novel AS events using a publicly available dataset on mouse mitochondrial proteome. We were particularly interested in understanding what fraction of novel peptides could be identified from translated mRNA sequences, and what factors affect the detectability of novel AS from MS/MS data. It is one of the first works in the field of connecting RNA-Seq data and MS/MS based proteomics data.

We observed that only a small proportion of novel RNA-Seq derived AS events could be validated by MS/MS spectra. Additionally, most of the novel peptides that we were able to identify could also be identified by searching against existing translated EST sequence databases. The smaller number of identified novel peptides than expected based on *in silico* analysis can in part be explained by lower abundance of the corresponding protein forms (below the range of detection of the mass spectrometer). It has also being suggested that only a small fraction of the alternative splice forms observed in the transcriptome data are functional and contribute to the diversity of the proteome [24,25]. This fundamental question should be addressed in future work.

This work has actually pointed out that current attempt to validate genome annotation based on proteomic data still has a lot of obstacles, both in data extraction and computational method. However, current method could still confidently identify some novel AS events from the combination of RNA-Seq data and proteomics data. Therefore, this work represents an effort of trying to find proteomic validation for genome annotation, which is very promising. Although the data used and approaches taken in this study are primary, and the results are not very promising, it may lead into the direction of addressing similar goals (e.g., more accurate gene annotation with the aid of proteomic data) using more diverse sets of matching RNA-Seq and proteomic data in the future.

## Authors’ contributions

KN and AIN conceived the project. KN conducted the analysis. KN and AIN wrote the paper. All authors contributed to the underlying ideas of the method and the analysis. All authors read and approved the final manuscript.

## Competing interests

No competing interests declared.

## References

[B1] Aebersold R Mann M Mass spectrometry-based proteomics Nature 2003 422 6928 198 207 10.1038/nature01511 12634793

[B2] Nesvizhskii AI Vitek O Aebersold R Analysis and validation of proteomic data generated by tandem mass spectrometry Nat Methods 2007 4 10 787 797 10.1038/nmeth1088 17901868

[B3] Nesvizhskii AI Vitek O Aebersold R Analysis and validation of proteomic data generated by tandem mass spectrometry Nature Methods 2007 4 10 787 797 10.1038/nmeth1088 17901868

[B4] Wu L Hwang SI Rezaul K Lu LJ Mayya V Gerstein M Eng JK Lundgren DH Han DK Global survey of human T leukemic cells by integrating proteomics and transcriptomics profiling Mol Cell Proteomics 2007 6 8 1343 1353 10.1074/mcp.M700017-MCP200 17519225

[B5] Cox J Mann M MaxQuant enables high peptide identification rates, individualized p.p.b.-range mass accuracies and proteome-wide protein quantification Nat Biotechnol 2008 26 12 1367 1372 10.1038/nbt.1511 19029910

[B6] Slebos RJ Brock JW Winters NF Stuart SR Martinez MA Li M Chambers MC Zimmerman LJ Ham AJ Tabb DL Evaluation of Strong Cation Exchange versus Isoelectric Focusing of Peptides for Multidimensional Liquid Chromatography-Tandem Mass Spectrometry J Proteome Res 2008 10.1021/pr8004666 PMC2669493 18939861

[B7] Nesvizhskii AI Roos FF Grossmann J Vogelzang M Eddes JS Gruissem W Baginsky S Aebersold R Dynamic spectrum quality assessment and iterative computational analysis of shotgun proteomic data: toward more efficient identification of post-translational modifications, sequence polymorphisms, and novel peptides Mol Cell Proteomics 2006 5 4 652 670 16352522 10.1074/mcp.M500319-MCP200

[B8] Ning K Fermin D Nesvizhskii AI Computational analysis of unassigned high quality MS/MS spectra in proteomic datasets Proteomics 2010 20455209 10.1002/pmic.200900473 PMC3517130

[B9] Edwards NJ Novel peptide identification from tandem mass spectra using ESTs and sequence database compression Mol Syst Biol 2007 3 102 17437027 10.1038/msb4100142 PMC1865584

[B10] Kersey PJ Duarte J Williams A Karavidopoulou Y Birney E Apweiler R The International Protein Index: an integrated database for proteomics experiments Proteomics 2004 4 7 1985 1988 10.1002/pmic.200300721 15221759

[B11] Castle JC Zhang C Shah JK Kulkarni AV Kalsotra A Cooper TA Johnson JM Expression of 24,426 human alternative splicing events and predicted cis regulation in 48 tissues and cell lines Nat Genet 2008 40 12 1416 1425 10.1038/ng.264 18978788 PMC3197713

[B12] Wang ET Sandberg R Luo S Khrebtukova I Zhang L Mayr C Kingsmore SF Schroth GP Burge CB Alternative isoform regulation in human tissue transcriptomes Nature 2008 456 7221 470 476 18978772 10.1038/nature07509 PMC2593745

[B13] Fermin D Allen BB Blackwell TW Menon R Adamski M Xu Y Ulintz P Omenn GS States DJ Novel gene and gene model detection using a whole genome open reading frame analysis in proteomics Genome Biol 2006 7 4 R35 16646984 10.1186/gb-2006-7-4-r35 PMC1557991

[B14] Chang KY Georgianna DR Heber S Payne GA Muddiman DC Detection of Alternative Splice Variants at the Proteome Level in Aspergillus flavus J Proteome Res10.1021/pr900602d 20047314

[B15] Nesvizhskii AI Roos FF Grossmann J Vogelzang M Eddes JS Gruissem W Baginsky S Aebersold R Dynamic spectrum quality assessment and iterative computational analysis of shotgun proteomic data - Toward more efficient identification of post-translational modifications, sequence polymorphisms, and novel peptides Molecular & Cellular Proteomics 2006 5 4 652 670 10.1074/mcp.M500319-MCP200 16352522

[B16] Boguski MS Lowe TM Tolstoshev CM dbEST--database for "expressed sequence tags" Nat Genet 1993 4 4 332 333 10.1038/ng0893-332 8401577

[B17] Aaronson JS Eckman B Blevins RA Borkowski JA Myerson J Imran S Elliston KO Toward the development of a gene index to the human genome: an assessment of the nature of high-throughput EST sequence data Genome Res 1996 6 9 829 845 10.1101/gr.6.9.829 8889550

[B18] Mortazavi A Williams BA McCue K Schaeffer L Wold B Mapping and quantifying mammalian transcriptomes by RNA-Seq Nat Methods 2008 5 7 621 628 10.1038/nmeth.1226 18516045 PMC13303166

[B19] Mo F Hong X Gao F Du L Wang J Omenn GS Lin B A compatible exon-exon junction database for the identification of exon skipping events using tandem mass spectrum data BMC Bioinformatics 2008 9 1 537 19087293 10.1186/1471-2105-9-537 PMC2636810

[B20] Power KA McRedmond JP de Stefani A Gallagher WM Gaora PO High-throughput proteomics detection of novel splice isoforms in human platelets PLoS One 2009 4 3 e5001 19308253 10.1371/journal.pone.0005001 PMC2654914

[B21] Wang Z Gerstein M Snyder M RNA-Seq: a revolutionary tool for transcriptomics Nat Rev Genet 2008 10.1038/nrg2484 PMC2949280 19015660

[B22] Trapnell C Pachter L Salzberg SL TopHat: discovering splice junctions with RNA-Seq Bioinformatics 2009 19289445 10.1093/bioinformatics/btp120 PMC2672628

[B23] Desiere F Deutsch EW King NL Nesvizhskii AI Mallick P Eng J Chen S Eddes J Loevenich SN Aebersold R The PeptideAtlas project Nucleic Acids Res 2006 34 Database issue D655 658 16381952 10.1093/nar/gkj040 PMC1347403

[B24] Birzele F Csaba G Zimmer R Alternative splicing and protein structure evolution Nucl Acids Res 2008 36 2 550 558 18055499 10.1093/nar/gkm1054 PMC2241867

[B25] Melamud E Moult J Stochastic noise in splicing machinery Nucl Acids Res 2009 37 14 4873 4886 19546110 10.1093/nar/gkp471 PMC2724286

[B26] Craig R Beavis RC TANDEM: matching proteins with tandem mass spectra Bioinformatics 2004 20 9 1466 1467 10.1093/bioinformatics/bth092 14976030

[B27] MacLean B Eng JK Beavis RC McIntosh M General framework for developing and evaluating database scoring algorithms using the TANDEM search engine Bioinformatics 2006 22 22 2830 2832 10.1093/bioinformatics/btl379 16877754

[B28] Pagliarini DJ Calvo SE Chang B Sheth SA Vafai SB Ong SE Walford GA Sugiana C Boneh A Chen WK A mitochondrial protein compendium elucidates complex I disease biology Cell 2008 134 1 112 123 18614015 10.1016/j.cell.2008.06.016 PMC2778844

[B29] Maglott D Ostell J Pruitt KD Tatusova T Entrez Gene: gene-centered information at NCBI Nucleic Acids Res 2007 35 Database issue D26 31 17148475 10.1093/nar/gkl993 PMC1761442

[B30] Karolchik D Baertsch R Diekhans M Furey TS Hinrichs A Lu YT Roskin KM Schwartz M Sugnet CW Thomas DJ The UCSC Genome Browser Database Nucleic Acids Res 2003 31 1 51 54 12519945 10.1093/nar/gkg129 PMC165576

[B31] Altschul SF Madden TL Schaffer AA Zhang J Zhang Z Miller W Lipman DJ Gapped BLAST and PSI-BLAST: a new generation of protein database search programs Nucleic Acids Res 1997 25 17 3389 3402 9254694 10.1093/nar/25.17.3389 PMC146917

[B32] Old WM Meyer-Arendt K Aveline-Wolf L Pierce KG Mendoza A Sevinsky JR Resing KA Ahn NG Comparison of Label-free Methods for Quantifying Human Proteins by Shotgun Proteomics Mol Cell Proteomics 2005 4 10 1487 1502 10.1074/mcp.M500084-MCP200 15979981

[B33] Zybailov B Coleman MK Florens L Washburn MP Correlation of relative abundance ratios derived from peptide ion chromatograms and spectrum counting for quantitative proteomic analysis using stable isotope labeling Analytical Chemistry 2005 77 19 6218 6224 10.1021/ac050846r 16194081

